# Selenium effectively inhibits 1,2-dihydroxynaphthalene-induced apoptosis in human lens epithelial cells through activation of PI3-K/Akt pathway

**Published:** 2011-07-21

**Authors:** Xiangjia Zhu, Kun Guo, Yi Lu

**Affiliations:** 1 Department of Ophthalmology, Eye & ENT Hospital, Fudan University, Shanghai, P.R. China; 2 Liver Cancer Institute, Zhongshan Hospital, Fudan University, Shanghai, P.R. China

## Abstract

**Purpose:**

To investigate whether activation of the phosphatidylinositol 3-kinase (PI3-K)/protein kinase B (Akt) pathway was necessary for selenium in protecting human lens epithelial cells (hLECs) from 1,2-dihydroxynaphthalene (1,2-DHN)-induced apoptosis. In addition, we studied the link between heat shock protein 70 (HSP70) expression and Akt phosphorylation in selenium-induced cell protection.

**Methods:**

Cell viabilities were assessed by Cell Counting Kit-8 (CCK-8) kit and trypan blue exclusion. The effect of sodium selenite on Akt phosphorylation was studied. After the pretreatment with 30 μM of LY294002, a PI3-K/Akt pathway inhibitor, apoptosis was assessed by flow cytometry, protein levels of phospho-Akt and Akt were quantified by western blot, and cell localization of phospho-Akt was determined by immunofluorescence staining. Time-course effect of sodium selenite on HSP70 expression was studied by reverse transcription polymerase chain reaction (RT–PCR) and western blot. Moreover, effect of LY294002 on HSP70 expression was also examined.

**Results:**

Our data showed that sodium selenite increased cell viabilities and prevented 1,2-DHN-induced apoptosis through phosphorylation and nuclear translocation of Akt. Furthermore, pretreatment of LY294002 inhibited the phosphorylation of Akt. However, it failed to block the selenium-induced upregulation of HSP70.

**Conclusions:**

The activation of PI3-K/Akt pathway was necessary for selenium in protecting hLECs from 1,2-DHN-induced apoptosis. However, this pathway was not involved in the selenium-induced upregulation of HSP70.

## Introduction

The anterior surface of the human lens is covered by lens epithelium. Cells near the lens equator divide and differentiate into lens fibers, and those in the central region protect the underlying fibers from injury and oxidative insult. Damage to the lens epithelium accumulates throughout a lifetime, leading to the apoptosis and inevitable reduction of lens epithelium density. Consequently, this diminishes the protective effect of the central epithelium over the underlying fibers [1]. Li et al. [2] have shown that apoptosis in the lens epithelium may be a common cellular basis for non-congenital cataract formation, and blocking apoptosis may prevent cataract formation. Charakidas et al. [3] also suggested that the accumulation of small-scale apoptotic epithelial losses during lifetime alters the lens fiber formation and homeostasis, resulting in a loss of lens transparency eventually. Therefore, it is important to develop protective strategies for apoptosis of human lens epithelial cells (hLECs).

Selenium is an essential trace element for humans. It has been recently recognized as a longevity-related element for some age-related diseases [4,5] due to its anti-oxidative properties. Selenium is in the active center of glutathione peroxidase (GPx), which protects membrane lipids and macromolecules from peroxide-induced oxidative damage. Moreover, it is required for the catalytic activation of other important anti-oxidant proteins in mammals, such as thioredoxin reductase and several other selenoproteins [6].

Recent studies have shown that selenium can also protect some cell lines against damages from free radicals and oxidative stress by suppressing apoptosis through the activation of phosphatidylinositol 3-kinase (PI3-K)/protein kinase B (Akt) pathway [6-8]. Akt, a serine threonine kinase, plays a critical role in regulating survival responses in many cells, such as neuronal cells [9], HT1080 cells (fibrosarcoma), and 3T3-L1 (rat adipocyte) [7]. As a downstream effector of PI3-K, phosphorylated Akt maintains cell survival and prevents apoptosis by inactivating several apoptosis effectors.

Our preliminary data showed that supplementation of selenium could impede the development of naphthalene cataract in adult rats by attenuating the oxidative stress in the lens (unpublished data). However, its underlying mechanisms remain unclear. One possible explanation might be the anti-apoptotic effect of selenium on lens epithelial cells. In the current study, we aimed to investigate whether selenium had this protective effect on hLECs through activation of PI3-K/Akt pathway. Moreover, we used 1,2-dihydroxynaphthalene (1,2-DHN) as an apoptosis-inducing agent in the present study, which is the metabolite of naphthalene in the lens and can subsequently auto-oxidized to 1,2-naphthaquinone and hydrogen peroxide [10,11].

Studies have also shown that selenium can upregulate the expression of heat-shock protein 70 (HSP70) under certain circumstances. For example, selenium mitigates the oxidative damage in fincoal-type fluorosis by increasing the expression of HSP70 [12]. This upregulation of HSP70 also plays a modulatory role against cerebral ischemia-induced neuronal damage in rat hippocampus [13]. Therefore, we evaluated whether selenium could increase the expression of HSP70 and the relationship between HSP70 expression and Akt phosphorylation in selenium-induced hLECs protection.

## Methods

### Materials

Sodium selenite was kindly provided by Professor Jinsong Zhang (School of Chemistry and Material Science, University of Science and Technology of China, Hefei, Anhui, P.R. China) as a gift. 1,2-DHN was purchased from Sigma (St. Louis, MO). LY294002, anti-Akt and anti-phospho-Akt (Ser473) antibodies were purchased from Cell Signaling Technology Inc. (Beverly, MA). Anti-HSP70 monoclonal antibody was obtained from Stressgen Bioreagents (Ann Arbor, MI). Horseradish peroxidase (HRP)-conjugated antibody against human β-actin was purchased from KangChen Bio-tech Inc. (Shanghai, China). Cell counting kit-8 (CCK-8) was obtained from Dojindo Laboratories (Kumamoto, Japan). Annexin V/Prodidium Iodide (PI) kit was purchased from Bender Medsystems (Vienna, Austria). Trizol reagent was obtained from Invitrogen Corporation (Camarillo, CA), and RevertAid first strand cDNA synthesis kit was purchased from Fermentas International Inc. (Ontario, Canada). In addition, 2× PCR Master Mix was purchased from Lifefeng Biotech Co., Ltd (Shanghai, China).

### Cell culture of hLECs

Briefly, hLECs (SRA01/04) were cultured in modified DMEM medium (high glucose) supplemented with 50 mg/l gentamicin and 10% heat-inactivated fetal bovine serum. Cells were maintained at 37 °C in a humidified atmosphere of 5% CO_2_ untill the semi-confluent monolayers were obtained.

### Cell viability analysis

Previous studies have revealed that the beneficial concentration of sodium selenite for cell culture varies from 2 ng/ml [8] to 3 μM (518.8 ng/ml) [7]. However, no data have reported a suitable concentration of sodium selenite for hLECs. Therefore, the optimum concentrations of sodium selenite and 1, 2-DHN were first optimized using CCK-8 assay and trypan blue exclusion. Briefly, hLECs were cultured in 96-well plates and treated with broad range of each reagent for 24 h. Subsequently, 10 µl of CCK-8 solution was added to each well, and the cells were maintained at 37 °C for another 2 h. Then the absorbance of samples was measured at a wavelength of 450 nm.

For trypan blue assay, 90 μl of cell suspension (1×10^6^ cells/ml) and 10 μl of 0.4% trypan blue were gently mixed. Then, 10 μl of stained cells were placed on a hemocytometer, and the numbers of viable (unstained) and dead (stained) cells were counted within 3 min. The percentage of viable cells was calculated accordingly.

Morphological changes of cells were observed under an inverted microscope (Olympus CK-30, Tokyo, Japan). Each experiment was done in triplicate.

### Flow cytometry

Cells were cultured in 6-well plates at a density of 1×10^6^ cells/well. After the treatment of sodium selenite and 1,2-DHN at desired concentrations and appropriate time periods, both floating and attached cells were collected. Then the cell pellets were washed twice with ice-cold phosphate buffered saline (PBS), resuspended in 200 μl of 1× binding buffer containing Annexin V (1:50 according to the manufacturer's instruction) and 40 ng/sample PI, and incubated at 37 °C for 15 min in the dark. Subsequently, the number of viable, apoptotic, and necrotic cells was quantified by flow cytometer (Becton Dickinson Company, Franklin Lakes, NJ).

### Western blot

To investigate the differentially expressed proteins in selenite-treated hLECs, cells were washed with PBS and lysed in 100 μl of lysis buffer (50 mM Tris-HCl, pH 7.5, 150 mM NaCl, 1 mM EDTA, 1% Triton X-100, 2.5 mM sodium pyrophosphate, 1 mM glycerophosphate, 1 mM Na_3_VO_4_ and 1 mM PMSF). Cell lysates were centrifuged at 4 °C 15,000× g for 10 min, and the supernatant was collected. Total protein content was determined by a Bio-Rad protein assay kit (Milan, Italy). For western blot, 30 μg of protein extracts were resolved by SDS–PAGE using 10% polyacrylamide gels and transferred to a PVDF membrane. Membranes were probed with antibodies against human Akt (1:1,000), phospho-Akt Ser473 (1:1,000), HSP70 (1:1,000), and β-actin (1:5,000), respectively. Then the membranes were incubated with species-specific secondary antibodies. Immunodetection was performed using standard ECL, and relative band intensities were quantified by ImageJ software (Image Processing and Analysis in Java).

### Immunofluorescence

One drop of cell suspension (2×10^4^ cells/ml) was dripped onto the coverslip at the bottom of a culture dish and cultured up to semi-confluency. After the treatment of sodium selenite and 1,2-DHN at desired concentrations and appropriate time periods, cells were rinsed with PBS, and then they were fixed with 4% paraformaldehyde. Subsequently, cells were permeabilized with 0.03% Triton X-100 in PBS for 10 min. To minimize the non-specific binding, fixed cells were blocked in 5% goat serum for 20 min. Cells were incubated with anti-phospho-Akt antibody (1:25 diluted in TBST [1× TBS, 5% BSA, and 0.1% Tween-20]) at 4 °C for 12–16 h. After the primary antibody labeling, cells were rinsed with PBS and incubated with goat anti-rabbit secondary antibody at room temperature for 30 min. Cells were then treated with 0.1 mg/ml RNAase at room temperature for 30 min and incubated in 5 μg/ml propidium iodide (PI; DNA stain) at 4 °C for 10 min. Finally, after rinsing with PBS, cells were mounted with glycerol and sealed with nail polish. Slides were examined under a confocal laser microscope (TCS SP2; Leica Microsystems, Wetzlar, Germany).

### RT–PCR (reverse transcription polymerase chain reaction)

Total RNA was extracted by using Trizol reagent according to the manufacturer’s instruction. Quantification and purity of total RNA was assessed by A260/A280 absorption. A total of 2 μg RNA was reversely transcribed with a first strand cDNA synthesis kit. Specific primers for human *HSP70* (forward: 5′-TGT TCC GTT TCC AGC CCC CAA-3′ and reverse: 5′-GGG CTT GTC TCC GTC GTT GAT-3′) were designed using Primer Premier 5.0 software, and glyceraldehyde 3-phosphate dehydrogenase (*GAPDH*, forward: 5′-CCA TGT TCG TCA TGG GTG TGA ACC A-3′ and reverse: 5′-GCC AGT AGA GGC AGG GAT GAT GTT C-3′) was selected as the housekeeping gene. PCR amplification was performed in a 20-μl system containing 10 pmol of primers. Following a denaturing step at 94 °C for 5 min, PCR reaction was performed with 30 cycles at a melting temperature of 94 °C for 30 s, an annealing temperature of 55 °C for 30 s, and an extension temperature of 72 °C for 30 s. Several experiments were performed to optimize the RT–PCR conditions. By using the optimized condition, neither human *HSP70* nor *GAPDH* reached the plateau after 30 cycles of amplification. Finally, an extra extension step at 72 °C for 7 min was preformed. The amplicons were analyzed on a 1.5% agarose gel by electrophoresis. The relative band intensities were quantified by ImageJ software (Image Processing and Analysis in Java).

### Statistical analysis

**Figure 1:**
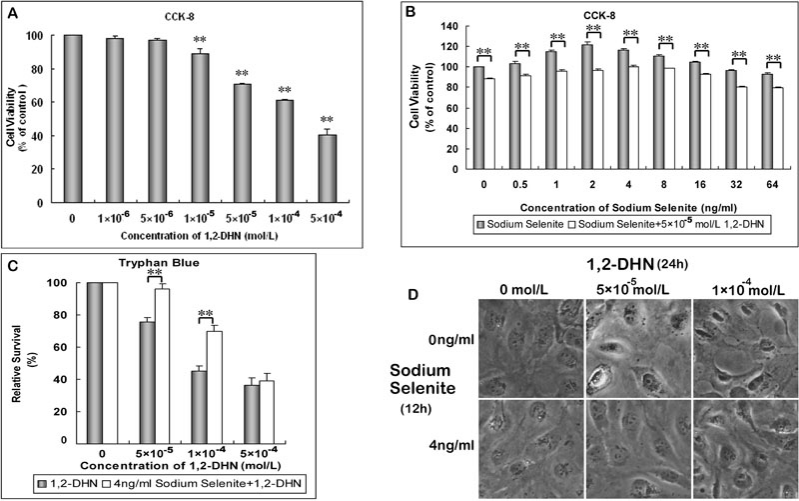
Sodium selenite increased cell viability and protected hLECs from 1,2-DHN-induced cell death. **A**: Cultured hLECs were treated with 1,2-DHN at different concentrations for 24 h. Cell viabilities were assayed using the CCK-8 kit. **B**: Cultured hLECs were either treated with sodium selenite alone at different concentrations for 12 h or given a combined treatment with 5×10^−5^ M 1,2-DHN for 24 h. **C**: Cultured hLECs were treated with 1,2-DHN alone at different concentrations for 24 h or given a pretreatment with 4 ng/ml sodium selenite for 12 h before the 1,2-DHN treatment. The relative survival rate was estimated by tryphan blue exclusion assay. **D**: Inverted microscope images of hLECs treated with 1,2-DHN at two concentrations with or without 4 ng/ml sodium selenite pretreatment. (*p<0.05, **p<0.01) Each experiment was performed in triplicate.

All data were expressed as mean±SD, representing three independent experiments. Paired *t*-test was used in determining the differences between single and combined treatment groups (Figure 1B,C). Other data were evaluated using ANOVA (ANOVA), and multiple comparisons were done by the least significant difference (LSD) test. A p-value less than 0.05 was considered as statistically significant. Data were analyzed using the Stata 7.0 statistical program.

## Results

### Sodium selenite increased cell viability and protected hLECs from 1,2-DHN-induced cell death

Using the CCK-8 kit, we studied the viabilities of hLECs treated with 1,2-DHN at different concentrations (1×10^−6^, 5×10^−6^, 1×10^−5^, 5×10^−5^, 1×10^−4^, and 5×10^−4^ M). After a 24-h incubation, the cell viabilities started decreasing as 1,2-DHN concentration reached 1×10^−5^ M (p<0.01, according to ANOVA analysis and LSD test, Figure 1A). Therefore, 1,2-DHN at a concentration of 5×10^−5^ M was used for intervention in our study, by which the cell viabilities were significantly decreased (Figure 1A). However, not many cells were killed at this concentration (Figure 1C).

To determine the beneficial concentration of sodium selenite for hLECs, we first analyzed effects of sodium selenite on hLECs at different concentrations (0.5, 1, 2, 4, 8, 16, 32, 64, 160, 320, and 640 ng/ml) by flow cytometry. Data revealed that hLECs started apoptosis after 12-h incubation when the concentration of sodium selenite was above 32 ng/ml (data not shown). Furthermore, we evaluated the cell viabilities of cells merely treated with sodium selenite (0.5, 1, 2, 4, 8, 16, 32, and 64 ng/ml) for 12 h and compared them with those treated with selenium for 12 h and 5×10^−5^ M 1,2-DHN for 24 h. Results showed that hLECs viabilities were increased as the concentrations of sodium selenite were between 1 to 8 ng/ml compared with the controls (Figure 1B). However, the viabilities were decreased as the concentration of sodium selenite was 64 ng/ml (all p<0.01, according to ANOVA analysis and LSD test). Moreover, cell viabilities in all the groups were significantly decreased with a further 1,2-DHN treatment for 24 h (all p<0.01, according to the paired *t*-test, Figure 1B). However, compared with the merely 1,2-DHN treated groups (for example: 0 ng/ml sodium selenite pretreatment group), cells pretreated with 1 to 16 ng/ml sodium selenite demonstrated higher cell viabilities, whereas the highest viability was obtained from the group treated with 4 ng/ml sodium selenite (all p<0.01; according to ANOVA analysis and LSD test, Figure 1B). Therefore, this concentration of sodium selenite was selected for interventions in the subsequent experiments.

For tryphan blue exclusion assay, hLECs were either treated with 1,2-DHN alone at different concentrations for 24 h or pretreated with sodium selenite for 12 h before 1,2-DHN treatment. Figure 1C shows that 1,2-DHN-induced cell death at high concentrations was apparent after 24 h of incubation. However, it was mitigated if the cells were pretreated with sodium selenite. Figure 1D shows that sodium selenite pretreated cells demonstrated a more normal morphology.

### Sodium selenite induced Akt phosphorylation in a PI3-K/Akt pathway-dependent manner

**Figure 2:**
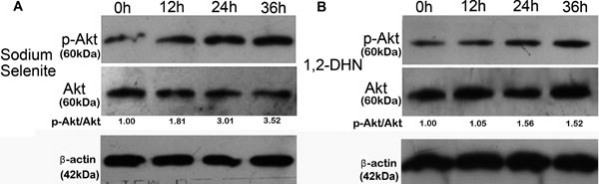
Sodium selenite induced Akt phosphorylation. **A**: Cultured hLECs were treated with 4 ng/ml sodium selenite for 12, 24, and 36 h, respectively. **B**: Cultured hLECs were treated with 5×10^−5^ M 1,2-DHN for 12, 24, and 36 h, respectively. Each experiment was performed in triplicate.

**Figure 3:**
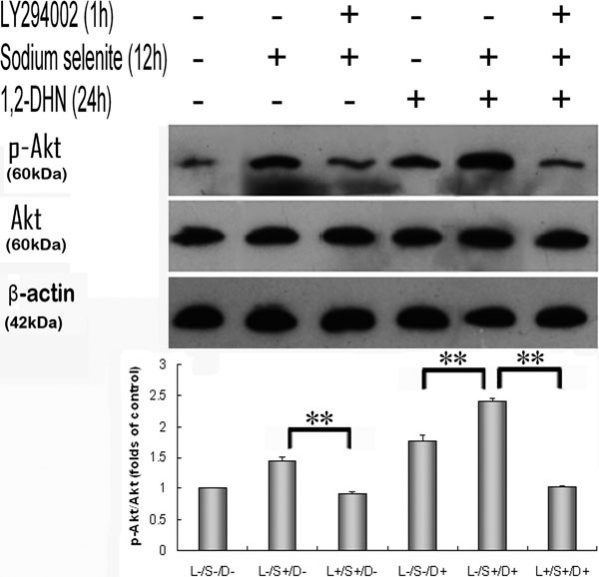
Sodium selenite induced Akt phosphorylation in a PI3-K-dependent manner either in the normal or 1,2-DHN stimulated state. The intervention duration of LY294002, sodium selenite and 1,2-DHN was 1, 12, and 24 h, respectively. Cells were harvested for western blot, and each experiment was performed in triplicate. (*p<0.05, **p<0.01).

**Figure 4:**
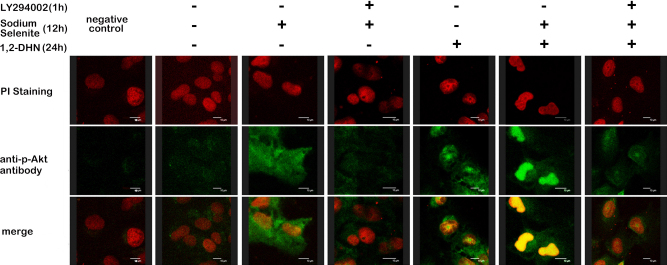
Immunofluorescence staining results also showed that sodium selenite induced Akt phosphorylation in a PI3-K-dependent manner either in the normal or 1,2-DHN stimulated state. The intervention duration of LY294002, sodium selenite and 1,2- DHN was 1, 12, and 24 h, respectively. It was noteworthy that pretreatment of 4 ng/ml sodium selenite apparently promoted Akt phosphorylation and nuclear translocation in hLECs treated with 5×10^−5^ M 1, 2-DHN. This effect was inhibited by 1-h pretreatment of 30 μM LY294002.

After exposure to 4 ng/ml sodium selenite for 12 h, Akt phosphorylation was significantly increased, and this effect retained in the next 24 h, whereas Akt phosphorylation was only slightly increased after the treatment of 5×10^−5^ M 1,2-DHN for 24 h as a response to the stimulation (Figure 2). LY294002 (30 μM), a specific inhibitor of PI3-K/Akt pathway, suppressed Akt phosphorylation induced by sodium selenite (Figure 3, column 1–3). Immunofluorescence staining also demonstrated the similar result. (Figure 4, column 2–4).

### Sodium selenite inhibited 1,2-DHN-induced apoptosis in hLECs through activation of PI3-K/Akt pathway

**Figure 5:**
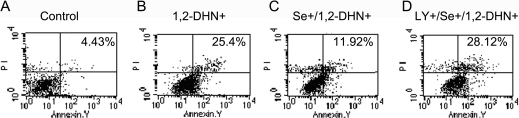
Sodium selenite inhibited 1,2-DHN-induced apoptosis in hLECs through activation of PI3-K/Akt pathway. Apoptosis was evaluated using Annexin V/PI staining and flow cytometry assay. Each experiment was performed in triplicate. **A**: Control group. Early apoptosis: 4.43±0.31%. **B**: Cultured hLECs were treated with 5×10^−5^ M 1,2-DHN for 24 h. Early apoptosis: 25.4±0.95%. **C**: Cultured hLECs were incubated with 4 ng/ml sodium selenite for 12 h before the 24-h treatment of 5×10^−5^ M 1,2-DHN. Early apoptosis: 11.92±0.86%. **D**: Cultured hLECs were first treated with 30 μM LY294002 for 1 h, and then the cells were incubated with 4 ng/ml sodium selenite for 12 h before the 24-h treatment of 5×10^−5^ M 1,2-DHN. Early apoptosis: 28.12±0.96%.

Flow cytometry assay showed that the cell apoptosis ratio was 25.4% after treatment of 5×10^−5^ M 1,2-DHN for 24 h, which was much higher than that of the normal control. Pretreatment of sodium selenite for 12 h effectively inhibited apoptosis, and the apoptosis ratio was decreased to 11.92%. Interestingly, LY294002 pretreatment (30 μM, 1 h) blocked the protective effect of sodium selenite, and the apoptosis ratio returned to 28.12% (all p<0.05; according to ANOVA analysis and LSD test, Figure 5).

Inhibition of apoptosis requires activation of functional survival proteins. Akt is such a protein, and its phosphorylated form can maintain cell survival and prevent apoptosis by inactivating several apoptosis effectors [7]. Our western blot results showed that phospho-Akt/Akt ratio of samples pretreated with sodium selenite was significantly increased compared with that of 1,2-DHN treated samples. Furthermore, such induction of Akt phosphorylation was again significantly inhibited by LY294002 pretreatment for 1 h (all p<0.01; according to ANOVA analysis and LSD test, Figure 3). Moreover, immunofluorescence staining further confirmed that phospho-Akt was mainly localized in the nucleus of cells pretreated with sodium selenite before 1,2-DHN treatment, which was obviously different from the cells treated with selenium only. LY294002 apparently blocked this selenium-induced Akt phosphorylation and translocation (Figure 4).

### Sodium selenite increased *HSP70* expression in hLECs

**Figure 6:**
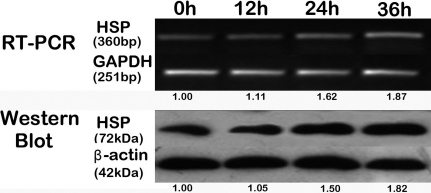
Sodium selenite induced HSP70 expression in a time-dependent manner. HLECs were treated with 4 ng/ml sodium selenite for 12, 24, and 36 h, respectively. The expression of *HSP70* at the mRNA and protein levels was quantified by RT–PCR and western blot analysis, respectively. Each experiment was performed in triplicate.

Previous studies have shown that HSP70 has protective effect on hLECs survival [14], and selenium can upregulate the expression of *HSP70* under certain circumstances [12,13]. To investigate whether HSP70 was necessary for selenium-induced hLECs protection, we assessed the time-course effect of sodium selenite on *HSP70* expression at the mRNA and protein levels using RT–PCR and western blot, respectively. Our results revealed that sodium selenite gradually increased the expression of *HSP70* in a time-dependent manner (Figure 6). The values underneath the bands represented the densitometric estimation of the relative band density. Furthermore, these values were calculated by normalizing *HSP70* to *GAPDH* or β-actin (*ACTB*) among various time points (the ratio at 0 h was set as baseline 1.0).

### Inhibition of PI3-K/Akt pathway did not affect the upregulated *HSP70* expression induced by sodium selenite

**Figure 7:**
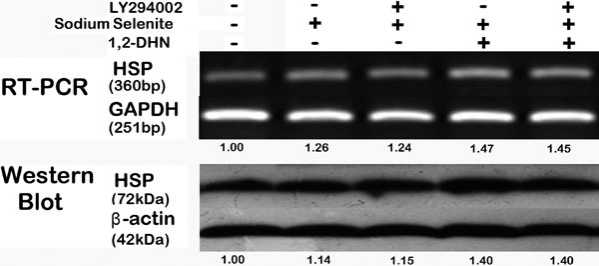
LY294002 did not affect the upregulated HSP70 expression induced by sodium selenite. The intervention duration of LY294002, sodium selenite and 1,2-DHN was 1, 12, and 24 h, respectively. The expression of *HSP70* at the mRNA and protein levels was quantified by RT–PCR and western blot analysis, respectively. Each experiment was performed in triplicate.

To explore the possibility of a link between the *HSP70* expression and Akt phosphorylation in the acquisition of hLECs protection induced by sodium selenite, we examined the effect of LY294002 (30 μM) on the selenite-induced upregulation of *HSP70* either under the normal or oxidant stimulated state. Although sodium selenite increased *HSP70* expression under both situations, pretreatment of LY294002 had no effect on this HSP70 upregulation (Figure 7). The values underneath the bands also represent the densitometric estimation of the relative density of the band. And each group was compared to the negative control, i.e., the LY294002(-)/Se(-)/1,2-DHN(-) group, which value was also set as the baseline 1.0.

## Discussion

Oxidative stress-induced apoptosis of lens epithelial cells is an early feature of age-related cataract, preceding the development of opacities in the lens fiber region [15,16]. Inhibition of this apoptosis effectively postpones the cataract development.

It has been reported that sodium selenite maintains HT1080 cells (fibrosarcoma) and 3T3-L1 (rat adipocyte) cells survival by activating the anti-apoptotic signal and blocking the apoptotic signal [7]. Moreover, it can also prevent secondary pathological events in an animal model of traumatic brain injury by blocking apoptotic neuron cell death [8]. However, this protective role of sodium selenite on hLECs remains unclear so far.

Regarding selenium, previous studies have been focused on selenite cataract, which is induced by giving a high-dose of selenite to suckling rats [17,18]. However, studies have also shown that selenium injections do not cause permanent cataract in rats after 18 days postpartum, suggesting that it has different effects on subjects at different ages [19,20]. Moreover, a few recent studies have investigated the protective effects of selenium against some types of cataract. It has been reported that a deficiency of selenium in the diet accelerates the progress of SDZ ICT 322 (indole-3-carboxylic acid scopoine ester, a selective hydroxytryptamine antagonist)-induced cataract [21], whereas administration of selenium and high dose vitamin E can protect lens from cisplatin-induced cataract [22]. Similarly, we used naphthalene cataract as a model to investigate the protective role of selenium on age-related cataract in our preliminary experiment. We found that the development of naphthalene cataract was impeded with supplementation of sodium selenite in the diet of adult Sprague Dawley rats via reducing the oxidative stress in the lens.

It is also known that the effect of selenium is dose-dependent. Some researchers have focused on the induction of apoptosis by toxic concentrations of selenium in cancer prevention and chemotherapy [23], while others have focused on the anti-oxidant effect of selenium at low concentrations [24]. In our present study, we first optimized the beneficial dose range of sodium selenite for hLECs. Within this range, it increased the viabilities of hLECs and promoted Akt phosphorylation in a PI3-K/Akt pathway-dependent manner. More importantly, selenite effectively inhibited the apoptosis induced by 1,2-DHN, a strong oxidant as well as the metabolite of naphthalene in the body, through activation of PI3-K/Akt pathway in hLECs. It suggested that sodium selenite, with a proper dose, could mitigate the oxidative stress in the lens through its anti-apoptotic effect. Immunofluorescence staining further confirmed that sodium selenite induced significant phosphorylation and nuclear translocation of Akt in hLECs in the presence of oxidative stress, which was apparently different from the unstimulated state.

This nuclear translocation of Akt noticed in this study may enhance the anti-apoptotic effect of selenium during oxidative stress. Caporali et al. [25] have reported that in cardiomyocytes, phosphorylation and nuclear translocation of Akt can promote the nuclear exclusion of Foxo-3a and Foxo-1, two members of Forkhead box, which promote cell death through activation of several Foxo-responsive genes, including the proapoptotic tumor necrosis factor-alpha (TNFα), Fas ligand, and bisindolyl maleimide-based, nanomolar protein kinase C inhibitors (Bim) [26-28]. Leinninger et al. [29] have shown that nuclear re-distribution of active Akt may be caused by insulin-like growth factor I (IGF-I)-mediated protection against high glucose-induced apoptosis. This nuclear re-distribution is followed by nuclear activation of survival transcription factors cyclic adenosine monophosphate (AMP) response element binding protein (CREB) as well as nuclear exclusion of Foxo-1 in dorsal root ganglion neurons. However, PI3-K/Akt pathway in the nucleus is more complicated and less known than it in the cytoplasm. Therefore, the downstream signaling mechanism of the Akt nuclear translocation noticed in this study should be evaluated in future investigations. It is notable that the anti-apoptotic function of PI3-K/Akt pathway can also promote the survival of a variety of tumor cells [30,31]. However, lens is an interesting organ, which is rarely or never affected by tumor due to its unique structure and surrounding immune environment. Therefore, activation of PI3-K/Akt pathway in lens may be safer than it in other cell lines.

Other studies have also shown that under certain circumstances, selenium can upregulate the expression of *HSP70*, which interacts with both the intrinsic and extrinsic pathways of apoptosis and inhibits cell death through chaperone dependent as well as independent activities [32]. We observed the similar function of selenium in hLECs both at the mRNA and protein levels. As we showed that activation of PI3-K/Akt pathway was involved in selenium-induced protection of hLECs, was there a link between this upregulated *HSP70* expression and the activation of PI3-K/Akt pathway? In renal cell carcinoma (RCC4) cells, PI3-K/Akt pathway inhibitor (LY294002) largely attenuates the increased *HSP70* expression toward heat treatment [33]. On the contrary, downstream induction of *HSP70* expression does not mediate the neuro-protective effect of Akt activation induced by thermal preconditioning in rat cerebellar granule neurons [9]. Our data also revealed that selenium treatment resulted in the upregulated *HSP70* expression. However, LY294002 exerted no effects on this induction of *HSP70*, suggesting that hLECs protection by selenium-induced PI3-K/Akt pathway activation was not mediated through the downstream induction of *HSP70* expression. Contradictory evidences concerning a link between Akt activity and *HSP70* expression may be due to cell-specific differences in the signaling pathways.

Although the downstream signaling mechanism of PI3-K/Akt pathway in this study remains unclear, our other findings are still promising. Due to the lack of clinical remedies for apoptosis of hLECs, a proper dose of selenium may provide basis in establishing a prospective strategy for cataract prevention.
